# Educational Attainment, School Quality and Midlife Cognitive Functioning: Differing Associations by Childhood Socioeconomic Status?

**DOI:** 10.1002/alz70860_101256

**Published:** 2025-12-23

**Authors:** Fabio Bolz, John Robert Warren, Eric Grodsky, Chandra Muller

**Affiliations:** ^1^ University of Minnesota, Minneapolis, MN, USA; ^2^ University of Wisconsin ‐ Madison, Madison, WI, USA; ^3^ University of Texas at Austin, Austin, TX, USA

## Abstract

**Background:**

Educational attainment has consistently been identified as an important determinant of later‐life cognitive functioning and recent studies, moreover, suggest that additional aspects of the educational process, particularly school quality, may be related to later‐life cognition. Many studies find a strong association between childhood socioeconomic status and later‐life cognition and that part of the association is mediated by educational attainment. What is not known, however, is whether the associations between the different aspects of the educational process and later‐life cognitive functioning vary by childhood SES: Is education more beneficial for the cognitive functioning of individuals from lower or higher socioeconomic backgrounds? The “cumulative advantage” or “resource multiplication” perspectives would hypothesize that individuals from higher socioeconomic backgrounds are likely to benefit more from higher school quality and a higher educational attainment. The “compensatory leveling” or “resource substitution” perspectives, in contrast, would hypothesize stronger effects for individuals from lower socioeconomic backgrounds.

**Method:**

Using long‐spanning nationally representative data from the U.S. High School & Beyond (HSB) cohort study, we examine whether the associations between school quality, educational attainment, and midlife cognitive functioning differ by childhood SES. HSB has followed a nationally representative sample of ∼25,500 people from high school in 1980 through midlife in 2021‐22. HSB data from the 1980s (when sample members were adolescents) includes rich information about educational contexts, opportunities, and outcomes; early life socioeconomic and family circumstances; spatial location; and demographic group memberships. HSB data from 2021‐22 (when most were approaching age 60) include high quality measures of cognitive functioning. The findings will have implications for our understanding of the life course processes underlying disparities in later‐life cognitive functioning.

**Result:**

None yet

**Conclusion:**

None yet

